# Altered Spontaneous Brain Activity in Women During Menopause Transition and Its Association With Cognitive Function and Serum Estradiol Level

**DOI:** 10.3389/fendo.2021.652512

**Published:** 2021-05-11

**Authors:** Lemin He, Wei Guo, Jianfeng Qiu, Xingwei An, Weizhao Lu

**Affiliations:** ^1^ Department of Biomedical Engineering, College of Precision Instruments and Optoelectronics Engineering, Tianjin University, Tianjin, China; ^2^ Department of Radiology, Shandong First Medical University & Shandong Academy of Medical Sciences, Taian, China; ^3^ Academy of Medical Engineering and Translational Medicine, Tianjin University, Tianjin, China; ^4^ Department of Radiology, The Second Affiliated Hospital of Shandong First Medical University, Taian, China; ^5^ Science and Technology Innovation Center, Shandong First Medical University & Shandong Academy of Medical Sciences, Jinan, China

**Keywords:** functional magnetic resonance imaging, spontaneous brain activity, menopause, estradiol, ReHo

## Abstract

**Objective:**

Serum hormone deficiencies during menopause transition may affect spontaneous brain activity and global cognition. The purpose of this study was to explore the differences in spontaneous brain activity between premenopausal and perimenopausal women, and to investigate the associations between spontaneous brain activity, serum hormone levels and global cognition.

**Methods:**

Thirty-two premenopausal women (47.75 ± 1.55 years) and twenty-five perimenopausal women (51.60 ± 1.63 years) underwent resting-state functional MRI (fMRI) scan. Clinical information including Mini-Mental State Examination (MMSE), levels of estradiol (E2), free testosterone, progesterone, prolactin, follicle-stimulating hormone and luteinizing hormone were measured. Regional homogeneity (ReHo) was used to evaluate spontaneous brain activity alterations between perimenopausal and premenopausal women. Correlation analysis was used to investigate the associations between brain functional alterations and clinical measures in perimenopausal group.

**Results:**

The results demonstrated increased ReHo value in the right lingual gyrus (LG) and decreased ReHo value in the right superior frontal gyrus (SFG) in perimenopausal women compared with premenopausal women. In perimenopausal group, ReHo of the right LG showed a negative correlation with level of E2 (r = -0.586, p = 0.002), ReHo of the right SFG showed a positive correlation with level of E2 (r = 0.470, p = 0.018) and MMSE (r = 0.614, p = 0.001).

**Conclusions:**

The results demonstrated that women approaching menopause suffered from altered functions in brain regions related to cognitive function, working memory, the results also revealed a direct association between levels of E2 and brain functions in perimenopausal women.

## Introduction

Perimenopause, also known as menopause transition, is the period between declined ovarian function and permanent cessation of menstruation in women (1). Erratic fluctuations in hormone levels lead to various physical manifestations which include hot flashes, night sweats, vasomotor dysfunction and vaginal dryness (2, 3). Indeed, perimenopause is regarded as “a window of vulnerability” for women (3). In addition, as the transition into menopause, many women experience cognition decline and memory loss (4). The most critical change during menopause transition is the fluctuation of hormone levels, especially the drop of estrogen levels (1, 5). There is growing evidence that serum hormone levels have effect on cognition and memory during menopause transition (6). Previous studies have investigated the effects of hormone therapy on cognition in women approaching menopause, and have found that women had better cognitive and memory performance in relevant tasks after hormone therapy (7). Hormones act throughout several cellular and molecular processes which can alter structure and function of the central nervous system *via* hormone receptors (1, 8). In the brain, hormone receptor expressions have been found in the cerebral cortex and limbic systems (8, 9). Hormones and neurosteroids also play critical roles in neural plasticity in the brain as well (10, 11).

In the recent decade, neuroimaging techniques have been applied to investigate alterations of the brain in women during menopause transition (1, 10, 12–14). Functional magnetic resonance imaging (fMRI), as a popular tool in neuroscience, has been used to study brain functional changes in women during menopause transition (1, 12). Specifically, fMRI has been applied to evaluate therapeutic effect of hormone therapy on cognitive function of women during menopause transition and have revealed that women during menopause transition may benefit from certain hormone therapy in terms of cognitive control, verbal and working memory (14–16). In addition, fMRI studies have also revealed functional activations in premenopausal and postmenopausal women related to sexual arousal (17–19). A previous study by our group revealed that perimenopausal women experienced altered intrinsic functional connectivity in regions related to sexual function (1).

For most fMRI studies related to women in menopause transition, the studies focused on brain functional changes under stimuli or after hormone therapy (14–19), few studies have focused on the hormonal fluctuations during menopause transition and its effect on spontaneous brain activity. Regional homogeneity (ReHo), one of the measures in resting-state fMRI, has been used to quantify the synchronization of a given voxel with its neighboring voxels (20). Unlike functional connectivity which involves in distant temporal correlations of fMRI signals, ReHo focuses on functional coherence of regional neural activity (21). Using ReHo analysis, researchers have identified brain functional alterations associated with Parkinson’s disease, depressive disorder, schizophrenia, etc. (22, 23). In this study, we aimed to find the differences in spontaneous brain activity between premenopausal and perimenopausal groups, and to explore the association between serum hormone levels and spontaneous brain activity in perimenopausal women. Regional homogeneity (ReHo) was used to quantify spontaneous brain activity, correlation analysis was used to evaluate the association between ReHo values, serum hormone levels and cognitive function.

## Materials and Methods

### Participants

This cross-sectional study received full approval from the Medical Ethics Committee of the Shandong First Medical University in accordance with the Declaration of Helsinki. All participants gave their written, informed consent before participating in this study. From June 2017 to January 2018, premenopausal and perimanopausal women were recruited by the Second Affiliated Hospital of Shandong First Medical University. Enrollment criteria for perimenopausal women included: (1) 45 - 55 years old, (2) more than 12 years of formal education, (3) right-handedness, (4) heterosexuality, (5) perimenopause was diagnosed according to the Stages of Reproductive Aging Workshop (STRAW) +10 staging system: persistent difference in consecutive menstrual cycle variable length was larger than 7 days or interval of amenorrhea was larger than 2 months (24). Enrollment criteria for premenopausal women included: (1) 45 - 55 years old, (2) more than 12 years of formal education, (3) right-handedness, (4) heterosexuality, (5) premenopause was diagnosed based on the diagnosis criterion of STRAW +10 staging system: having a regular ovulation day according to the rhythm method. Exclusion criteria for the enrolled women were: (1) history of psychiatry or neurological disorders, (2) hormone or steroid treatment in a month prior to the study, or oral contraceptive use in a month prior to the study, (3) use of antihistamines, ranitidine, black cohosh, or other drugs that modulate ovarian steroid secretion in a month prior to the study, (4) dysfunction of organs including heart, liver or kidney, (5) endocrine diseases, (6) premenstrual syndrome or premenstrual dysphoric disorder, (7) MRI contradictions. At last, 32 premenopausal women (47.75 ± 1.55 years) and 25 perimenopausal women (51.60 ± 1.63 years) were finally recruited. Upon enrollment, all subjects received a routine body examination including routine blood test, urine test, chest and abdominal CT, routine gynecological examination to rule out any possible effect on the bioavailability of steroids and brain function.

### Measurement of Cognitive Function and Serum Hormone Concentrations

Cognitive function was evaluated by the Mini-Mental State Examination (MMSE). In terms of serum hormones, the concentrations of six hormones included estradiol (E2), prolactin (PRL), luteinizing hormone (LH), follicle-stimulating hormone (FSH), free testosterone (free-T) and progesterone (P) were measured during the early follicular phase (3-5 days after menstrual onset). Participants were instructed to have a good night sleep before the test. The test was carried out between 8:30 and 9:30 a.m. Venous blood samples were obtained from all subjects *via* vein puncture. The levels of E2, PRL, LH, FSH, free-T and P were measured using chemiluminescent immunoassay method by E170 Immunology Analyzer (Roche, Brussels, Belgium).

### fMRI Acquisition and Processing

A 3.0T MR scanner (Discovery MR 750, GE, Milwaukee, US) with 8-channel head array coil was used to acquire fMRI data. Participants were scanned in a supine, head-first position with cushions on both sides and at top of the head to control head motion. T1-weighted structural images were obtained *via* 3D-BRAVO sequence with the following parameters: repetition time (TR) = 6.656 ms, echo time (TE) = 2.928 ms, inversion time = 450 ms, field of view (FOV) = 240 mm × 240 mm^2^, slice thickness = 1 mm, slice gap = 1 mm, matrix = 256 × 256, number of signal averages = 1, flip angle (FA) = 12°, and 176 sagittal slices. Before resting-state fMRI scan, participants were instructed to open their eyes, calm breathing, keep a clear consciousness and not to engage in any specific thinking activity. To acquire resting-state fMRI data, echo-planar imaging sequence was used with the following parameters: TR = 2000 ms, TE = 30 ms, FOV = 240 mm × 240 mm^2^, matrix = 64 × 64, slice thickness = 3.5 mm, slice gap = 1.2 mm, FA = 90°, scan duration = 480 s (240 volumes) and 33 axial slices.

Data Processing & Analysis for Brain Imaging (DPABI, http://rfmri.org/dpabi) was used for fMRI data preprocessing, regional homogeneity (ReHo) calculation and statistical analysis. Data preprocessing included the following steps: (1) The first 10 volumes of the fMRI data were removed to preserve steady-state data only. (2) The remaining fMRI volumes were corrected for timing differences and for head motion. Subjects with head motion more than 1 mm, head rotational motion larger than 1° or framewise displacement more than 0.5 mm were excluded from further analysis. 9 participants were excluded and they were contacted for fMRI rescans. Finally, all the enrolled women passed the thresholds. (4) The individual fMRI images were spatially registered to the Montreal Neurological Institute (MNI) standard space using Diffeomorphic Anatomical Registration Through Exponentiated Lie Algebra algorithm. (5) Nuisance covariates including head motion parameters, white matter and cerebro-spinal fluid signal were regressed out from each subject’s fMRI time series. (6) A band-pass filter (0.01-0.08 Hz) was used to reduce low-frequency drifts and high-frequency noise.

After data preprocessing, ReHo map for each woman was calculated. In the present study, ReHo value of each voxel was calculated as the Kendall’s coefficient concordance (KCC) of this voxel with its adjacent 26 voxels (20). ReHo value close to 1 means a given voxel and its adjacent voxels are more consistent, vice versa (20). Then, ReHo maps were spatially smoothed with a Gaussian kernel (full width half maximum = 6 mm).

### Statistical Analysis

SPSS 20.0 was used for statistical analysis. Independent t-test was used for comparisons of age, education and MMSE between premenopausal and perimenopausal women. Mann-Whitney U test was used to compare serum hormone levels between the two groups. The threshold for significance was set at p < 0.05.

Comparison of ReHo maps between premenopausal and perimenopausal women was carried out using the statistical module of DPABI. Specifically, general linear model was used to detect whether there were differences in ReHo maps between the two groups. Age was treated as a nuisance covariate and was adjusted using linear regression. Gaussian random field (GRF) correction with voxel level p < 0.001 and cluster level p < 0.05 (two-tailed) was used to control false positives. In addition, effect size (ES) calculated as Cohen’s d was used to evaluate statistical effect of the group analysis.

Associations between ReHo values and clinical measures (including MMSE, serum hormone concentrations) in perimenopausal group were evaluated by Pearson’s correlation analysis. Group analysis revealed several regions with significant differences in ReHo values between the two groups. Mean ReHo values were extracted from these regions in perimenopausal group. Pearson’s correlation analysis was conducted to investigate the association between mean ReHo values and serum hormone concentrations. In addition, relationship between ReHo values and MMSE was also assessed using Pearson’s correlation analysis. The threshold for statistical significance was set at p < 0.05.

## Results

### Demographic and Clinical Information

Demographic information and serum hormone concentrations of premenopausal and perimenopausal women are listed in **Table 1**. MMSE scores and six hormone levels were all in the normal range for premenopausal and perimenopausal women. In addition, increased levels of FSH and LH in perimenopausal women were observed, while decreased levels of PRL, E2, free-T and P in perimenopausal women were found compared with premenopausal women. Significant differences in age, MMSE, levels of PRL, FSH, LH, E2 and P were found between premenopausal and perimenopausal women. There were no significant statistical differences in education duration and level of free-T between the two groups.

**Table 1:** Demographic information and sex hormone levels between premenopausal (n = 32) and perimenopausal women (n = 25).

	Premenopausal women (n = 32)	Perimenopausal women (n = 25)	Normal range	P value
Age (years)^a^	47.75 ± 1.55	51.60 ± 1.63	–	<0.001^b^
Education (years)^a^	16.18 ± 1.31	15.84 ± 0.80	–	0.248^b^
MMSE^a^	29.28 ± 0.85	28.68 ± 1.28	27 - 30	0.038^b^
PRL (ng/ml)^a^	21.67 ± 17.71	12.93 ± 10.68	1.9-25	0.011^c^
FSH (mIU/ml)^a^	5.74 ± 4.21	52.63 ± 29.75	1.2-153	<0.0001^c^
LH (mIU/ml)^a^	7.31 ± 13.13	22.12 ± 11.32	1.1-77	0.0013^c^
E2 (pg/ml)^a^	109.70 ± 64.55	35.70 ± 22.70	20-400	<0.0001^c^
free-T (ng/dL)^a^	28.47 ± 16.35	19.13 ± 1.27	0-73	0.403^c^
P (ng/ml)^a^	2.37 ± 4.23	0.22 ± 0.06	0.1-24	0.001^c^

a Variables are presented as mean ± standard deviation.

b P value was calculated via independent t-test.

c P value was calculated via Mann-Whitney U test.

PRL, prolactin; FSH, follicle-stimulating hormone; LH, luteotropic hormone; E2, estradiol; free-T, free testosterone; P, progesterone.

### ReHo Changes

**Figure 1:**
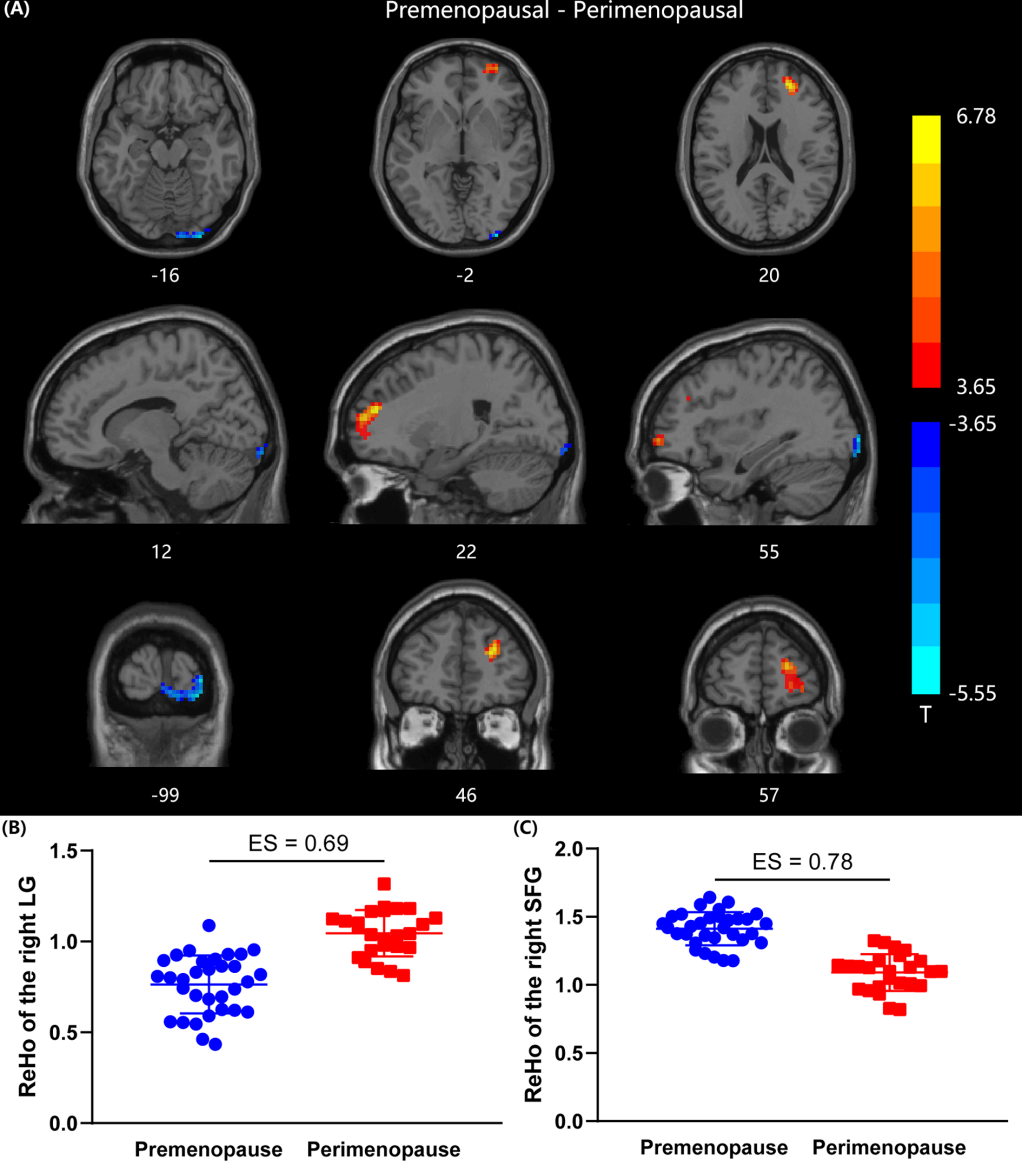
Comparison of ReHo values between premenopausal group (n =32) and perimenopausal group (n = 25). **(A)** Two sample t-map between premenopausal women and perimenopausal women. Gaussian random field correction (two tailed, voxel-level p < 0.001, cluster level p < 0.05) was used for multiple comparisons, colorbar indicates T-score. **(B)** Scatter plot of mean ReHo value in the right LG for the two groups. **(C)** Scatter plot of mean ReHo value in the right SFG for the two groups. LG, lingual gyrus; SFG, superior frontal gyrus; ES, effect size.

Compared with premenopausal women, perimenopausal women demonstrated increased ReHo in the right lingual gyrus (LG) (GRF corrected at voxel level p < 0.001 and cluster level p < 0.05, two-tailed, ES = 0.69). Perimenopausal women also showed decreased ReHo values in the right superior frontal gyrus (SFG) compared with premenopausal women (GRF corrected at voxel level p < 0.001 and cluster level p < 0.05, two-tailed, ES = 0.78). The differences in ReHo map between the two groups are shown in **Figure 1**. The brain regions with significant differences in ReHo are identified in **Table 2**.

**Table 2:** Brain regions with significant differences in ReHo values between premenopausal (n = 32) and perimenopausal women (n = 25) (Gaussian random field corrected at voxel level p < 0.001, cluster level p < 0.05, two-tailed).

Brain region	BA^a^	Cluster size^b^	MNI coordinates^c^			T value^d^	Types of ReHo change^e^
			X	Y	Z	
Right LG	17,18	79	33	-99	-3	-5.430	Premenopausal < Perimenopausal
Right SFG	9,10,46	197	21	48	21	6.776	Premenopausal > Perimenopausal

a BA represents Brodmann area, which is an atlas of the human cerebral cortex with 52 subregions.

b Cluster size represents number of voxels in the relevant clusters.

c MNI coordinates are the coordinates of voxels in the standard brain atlas provided by Montreal Neurological Institute.

d T value is the statistical value from independent t-test. In this column, T value represents T value of the peak voxel in the cluster.

e ReHo changes have two types, ReHo value of the premenopausal group is larger than ReHo value of the perimenopausal group, or ReHo value of the premenopausal group is smaller than ReHo value of the perimenopausal group.

ReHo, regional homogeneity; BA, Brodmann area; MNI, Montreal Neurological Institute; LG, lingual gyrus; SFG, superior frontal gyrus.

### Correlation Analyses

**Figure 2:**
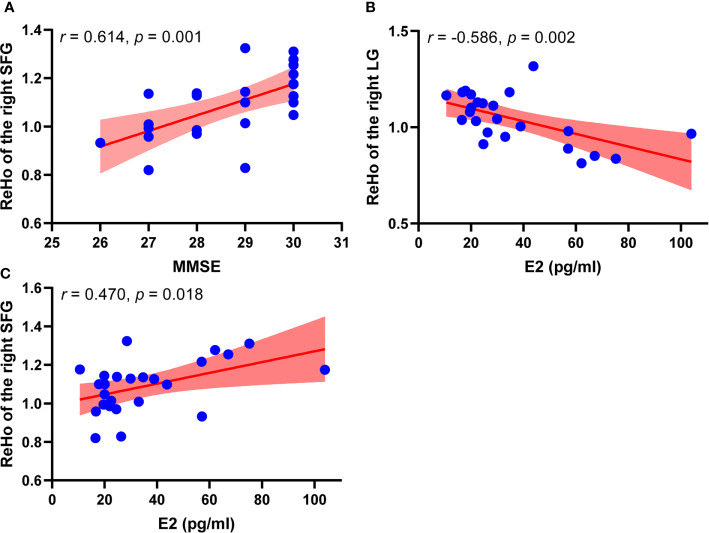
Correlation analysis results between ReHo values and clinical information in perimenopausal group (n = 25). **(A)** Scatter plots between ReHo value of the right SFG and MMSE in perimenopausal women. **(B)** Scatter plots between ReHo value of the right LG and level of E2 in perimenopausal women. **(C)** Scatter plots between ReHo value of the right SFG and level of E2 in perimenopausal women. MMSE, Mini-Mental State Examination, LG; lingual gyrus, E2, estradiol; ReHo, regional homogeneity; SFG, superior frontal gyrus.

Correlation analysis results are shown in **Figure 2**. In perimenopausal group, ReHo of the right SFG showed a positive correlation with MMSE (r = 0.614, p = 0.001). ReHo of the right LG showed a negative correlation with the level of E2 (r = -0.586, p = 0.002), ReHo of the right SFG showed a positive correlation with the level of E2 (r = 0.470, p = 0.018). However, ReHo values did not show significant correlations with other hormone levels. In addition, ReHo value of the right LG and ReHo of the right SFG did not have a significant correlation.

## Discussion

Although previously viewed as a reproductive and psychological transition, a recent state-of-the-art study has pointed out that perimenopause is largely a neurological transition in nature (25). Menopausal symptoms that emerge during perimenopause indicate disruptions in multiple hormone-regulated systems (25). The fluctuations of serum hormones including sex hormones, estrogens could affect the structure and function of the central nervous system through a network of hormone receptors (8, 9, 25). Therefore, it is of clinical importance to study brain function alterations, and to explore the association between serum hormone levels and brain functions in perimenopausal women.

For the enrolled participants in the current study, the concentrations of six hormones included E2, PRL, LH, FSH, free-T and P were measured to provide a general evaluation of endocrine status. Results indicated that concentrations of six hormones were all in the normal range for both premenopausal and perimenopausal group. However, perimenopausal women experienced increased levels of FSH and LH, and decreased levels of E2, PRL and P compared with premenopausal women. As is known, E2 and P are synthesized in the ovary, while PRL, FSH and LH are released by the pituitary gland (10, 26). In perimenopause, declined ovarian function results in the decreased release of E2 and P (1). As a result, the pituitary gland releases more FSH and LH to stimulate ovary to produce steroids (27). Different from FSH and LH, PRL secretion is not affected by the declined ovarian function (28). The decreased PRL level for perimenopausal women in this study may be a direct consequence of age-related processes in the lactotrophs (28). In addition, other factors involved in the regulation of PRL secretion, such as estrogens should also be considered (29).

In the field of neuroimaging, ReHo, an fMRI measure, has been widely used to evaluate spontaneous brain activity and brain functions at resting-state (20). In the present study, ReHo from resting-state fMRI was used to evaluate brain functional alterations in perimenopausal women, and correlation analysis was used to evaluate the relationship between serum hormone levels, cognitive function and spontaneous brain activity. The results demonstrated that perimenopausal women showed both decreased spontaneous brain activity and increased spontaneous brain activity in several brain regions compared with premenopausal women. E2 level had significant associations with ReHo of several brain regions related to cognitive function.

In this study, perimenopausal women showed decreased ReHo in the right SFG compared with premenopausal women. Furthermore, MMSE was positively correlated with ReHo of the right SFG. The SFG makes up about one third of the frontal lobe in the human brain. The SFG which includes the supplementary motor area, is involved in cognitive function and working memory (30). The decreased spontaneous brain activity in the right SFG in perimenopausal women might indicate that perimenopausal women had a greater chance of experiencing decreased cognitive function and decreased working memory than premenopausal women. SFG is also implicated in depression as a neuroimaging study has revealed that dysfunction in the SFG causes depression (31). The present finding might give potential explanation for depression in women during menopause transition. In addition, a previous voxel-based morphometry study by our group has found that perimenopausal women showed decreased gray matter volume in the right SFG compared with premenopausal women (10). There is a close relationship between structure and function of the human brain (32). Functional changes in the right SFG might have associations with structural changes in the right SFG in perimenopausal women.

The current study also revealed increased ReHo in the right LG for perimenopausal women compared with premenopausal women. The LG is part of the visual cortex which are mainly involved in visual information processing (33). However, in addition to visual function, the LG is also responsible for visual working memory processing (34, 35). Several fMRI studies have reported the role of the visual cortex in retaining visual working memory information and working memory consolidation (36, 37). Although the occipital and frontal lobe are anatomically distant, yet these two regions are highly integrated in function (38). The present findings might suggest that the LG in the occipital lobe and the SFG in the frontal lobe both experienced altered spontaneous brain activity in relation to E2 deficiency in women during menopause transition.

E2 is a form of estrogens which is implicated in numerous physiological processes (39). Studies have reported that E2 is implicated in cognitive function, mood regulation, learning, memory, etc. (39, 40). In addition, E2 plays a critical role in neurodegenerative diseases including Alzheimer’s disease, dementia and stroke (41). In human, the brain expresses high levels of E2 receptors in several brain regions (42).

Present findings revealed that serum E2 level had a negative correlation with brain activity in the right LG, and serum E2 level had a positive correlation with brain activity in the right SFG in perimenopausal women. There is evidence that E2 receptors are located in the frontal cortex (13). Previous studies have reported that E2 level had an association with activity of the frontal cortex during emotion regulation and sexual stimuli (12, 43). In line with previous findings, the positive correlation between E2 level and brain activity in the right SFG suggested that E2 might positively contribute to the function of the frontal cortical system in related tasks. Davis et al. have investigated the effects of sex hormones on visuospatial function and verbal fluency in women during menopausal transition and have found that hormone therapy was associated with decreased brain activity in the lingual gyrus and occipital gyrus (44, 45). Similar phenomenon has also been reported by Neele et al. (46). It was hypothesized that with increased serum E2 level, less neural recruitment was required for task completion with the same speed and accuracy. On the contrary, when the level of E2 decreased, more neurons were needed in the occipital lobe for task completion, therefore, perimenopausal women with declined E2 level showed increased brain activity in the right LG.

Several limitations need to be addressed for this study. Firstly, clinical information and fMRI data were collected at one single time point due to limited conditions. Secondly, relatively small sample size may reduce statistical power and conclusions drawn from the results, however, effect size by Cohen’s d revealed strong statistical differences, and multiple comparison correction methods enhanced reliability of the current study. Future studies will focus on a larger study sample size with more clinical information.

In conclusion, resting-state fMRI was used to assess the differences in spontaneous brain activity between premenopausal and perimenopausal women. Correlation analysis was used to evaluate the association between serum hormone concentrations, cognitive function and spontaneous brain activity. The results demonstrated alterations of spontaneous brain activity and functional compensation in perimenopausal women compared with premenopausal women. In addition, the results also suggested that estradiol level had correlations with several regions related to cognition and visual working memory. The findings highlighted the association between estradiol and brain functions in women during menopause transition and might be helpful in understanding functional changes in the brain of women during menopause transition.

## Data Availability Statement

The raw data supporting the conclusions of this article will be made available by the authors, without undue reservation.

## Ethics Statement

The studies involving human participants were reviewed and approved by Medical Ethics Committee of the Shandong First Medical University. The patients/participants provided their written informed consent to participate in this study.

## Author Contributions

LH and WG conceived the study, XA and WL designed the protocol. JQ conducted the statistical analyses. LH, JQ, and XA interpreted study findings and contributed to developing the manuscript. LH and WL wrote the first draft of the manuscript that was revised and approved by all authors. All authors contributed to the article and approved the submitted version.

## Funding

This work was supported by the Traditional Chinese Medicine Science and Technology Development Plan of Shandong Province (grant number 2019-0359), the Medicine and Health Science Development Plan of Shandong Province (202009040008), Key Research and Development Program of Shandong Province (grant number 2017GGX201010), Academic Promotion Program of Shandong First Medical University (grant number 2019QL009) and Taishan Scholars Program of Shandong Province (grant number TS201712065).

## Conflict of Interest

The authors declare that the research was conducted in the absence of any commercial or financial relationships that could be construed as a potential conflict of interest.
